# Right-Ventricle-Adjacent Mass: A Multimodality Imaging Approach to Diagnosis

**DOI:** 10.3390/diagnostics15243227

**Published:** 2025-12-17

**Authors:** Chirine Liu, Romain Van der Linden, Mohamed El Mallouli, Nasroola Damry, Georgiana Pintea Bentea

**Affiliations:** 1 Department of Cardiology, CHU Brugmann, 1020 Brussels, Belgium; 2 Department of Radiology, CHU Brugmann, 1020 Brussels, Belgium; 3 Department of Cardiology, CHU Namur Sainte Elisabeth, 5000 Namur, Belgium

**Keywords:** ventricular adjacent mass, pericardial cyst, multimodality imaging

## Abstract

We report the case of a 53-year-old male patient who presented to the cardiology department with presyncope and atypical chest pain. The transthoracic echocardiography revealed a homogeneous hypoechoic mass measuring 2.5 × 5.7 cm at the level of the anterolateral wall of the right ventricle. In order to further characterize the identified right-ventricle-adjacent mass, we performed a cardiac computed tomography, which confirmed the presence of a homogeneous hypodense mass with a single wall, without septation. Cardiac magnetic resonance imaging demonstrated a serous fluid mass capping the right atrium, right atrial appendage, and coronary sinus, without evidence of myocardial invasion. The multimodality imaging performed clarified the diagnosis of an uncomplicated pericardial cyst. The patient was managed conservatively with every 6 months echocardiographic evaluation. At a 2-year follow-up, he presented no recurrent symptoms, and the pericardial cyst maintained the same characteristics. The cornerstone of this case report was relying on multimodality imaging in order to characterize the adjacent cardiac mass and to arrive at the diagnosis of an uncomplicated pericardial cyst, which established the prognosis and management of the patient.

**Figure 1:**
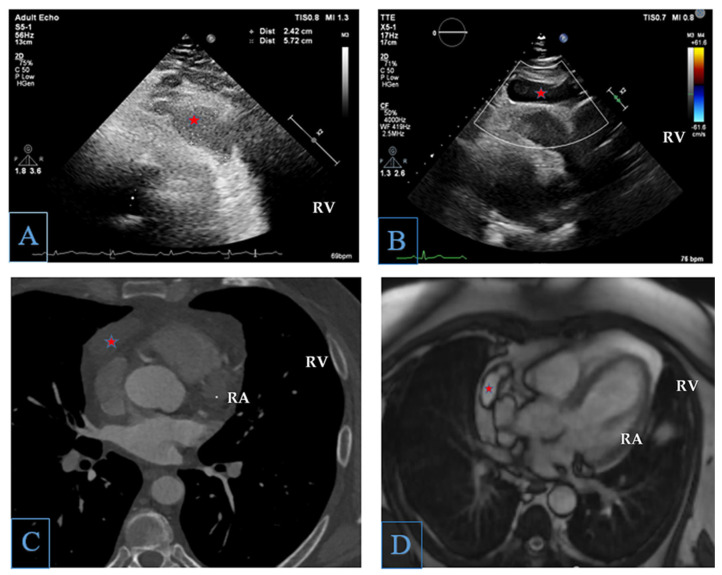
Panels (**A**,**B**): TTE, parasternal short axis showing a homogeneous hypoechoic mass (red star). Panel (**C**): cardiac CT, axial plane showing a homogeneous hypodense mass with a single wall, without septation (red star). Panel (**D**): cardiac MRI, axial view, T2−weighted sequences revealing a homogeneous mass with high signal intensity on T2−weighted sequences (red star). There was no evidence of late gadolinium enhancement. RA, right atrium, RV, right ventricle. We describe the case of a 53-year-old patient who presented for a cardiology consultation with an episode of presyncope and associated chest pain. He also reported progressive exertional dyspnea over the past year. His medical history was significant for arterial hypertension, obesity, and being a former smoker, the patient having successfully managed to quit 20 years prior. The electrocardiogram was unremarkable. The transthoracic echocardiography showed normal size and function of both the left and right cardiac chambers, with no valvular disease or pericardial effusion. However, during the echocardiography, a homogeneous hypoechoic mass measuring 2.5 × 5.7 cm was observed in contact with the anterolateral wall of the right ventricle (**A**,**B**), without any extrinsic compression. The blood work-up revealed inflammatory markers such as CRP and leucocyte count within normal range. Furthermore, the patient presented no documented history of pericarditis. In order to further characterize the identified right-ventricle-adjacent mass, we performed a cardiac computed tomography (CT), which confirmed the presence of a homogeneous hypodense mass adjacent to the pericardium, measuring 4.5 × 2.5 × 5.0 cm, with a single wall, without septation or calcifications (**C**). No significant coronary artery lesions were identified. Cardiac magnetic resonance imaging (MRI) demonstrated a mass measuring 4.5 × 2.0 × 5.2 cm capping the right atrium, right atrial appendage, and coronary sinus, without evidence of myocardial invasion (**D**). The mass presented high signal intensity on T2−weighted sequences and low intensity signal on T1−weighted sequences, suggesting a serous fluid nature of the mass, as such precluding the possibility of pericardial hematoma that usually presents high signal intensity on both T1− and T2−weighted sequences [1]. The unilocular isolated pericardial cyst in a patient without a history of traveling in an endemic area significantly decreased the likelihood of a hydatid cyst [2]. There was no late gadolinium enhancement, an MRI characteristic in line with an avascular structure, and the absence of a fibrous component. The overall characteristics of the observed pericardial mass excluded the diagnosis of pericardial abscess or tumor, such as mesothelioma, teratoma, or metastases [3]. Mesothelioma appears isointense on T1−weighted images and heterogeneous on T2−weighted sequences due to areas of necrosis, and it shows enhancement following gadolinium administration [4]. On the other hand, pericardial metastases present as multiple focal nodular lesions, pericardial thickening, or associated hemorrhagic pericardial effusion, which typically exhibit high signal intensity on T1−weighted sequences [5]. Furthermore, neither the cardiac MRI nor the cardiac CT demonstrated a communication between the mass and a cardiac cavity, thereby ruling out the hypothesis of a cardiac diverticulum [6]. In addition, the cardiac MRI failed to show a communication of the right-ventricle-adjacent mass with the pericardial sac, and the echocardiography revealed the lack of synchronous movement of the pericardial mass with breathing, effectively excluding the possibility of a pericardial diverticulum [6]. The multimodality imaging performed clarified the diagnosis of an uncomplicated pericardial cyst. Given that the patient presented with presyncope, a 24-h rhythm monitor was performed, and revealed no arrhythmias. Furthermore, there were no imaging indications of compression due to the pericardial cyst. In the end, the symptoms were not attributed to the cyst. The patient was managed conservatively with every 6 months echocardiographic evaluation. At 2 years follow-up, he presented no recurrent symptoms, and the pericardial cyst kept the same characteristics, including dimensional stability as assessed using echocardiography and cardiac CT scan. Pericardial cysts are rare. The incidence is 1 in every 100,000 patients [5]. The diagnosis is most often a serendipitous discovery, due to the low symptomatology. Symptoms are rare and may appear due to compression effects on adjacent structures; as such, habitually, the complications of a pericardial cyst depend on the size of the cyst [6]. Symptoms include cough, chest pain, dyspnea, and palpitations [1]. The most common origin is congenital, due to a failure of fusion of mesenchymal lacunae during the formation of the pericardial sac. It can also result from inflammatory situations (pericarditis, tuberculosis), traumatic situations, or iatrogenic causes (cardiac surgery). Current recommendations advocate for non-invasive surveillance of the cyst through serial cardiac CT or MRI at 1- to 2-year intervals. In symptomatic patients, when technically feasible, initial intervention is directed toward either percutaneous or endoscopic aspiration with subsequent ethanol sclerosis, or thoracoscopic surgical excision [7]. The primary aim of this paper was to demonstrate the value of multimodal imaging in characterizing the adjacent cardiac mass we identified, as its pathological implications and management differ substantially. Although echocardiography successfully detected the abnormality, it alone is insufficient to define the precise pathological entity. Ultimately, establishing an accurate diagnosis remains essential for determining the pericardial mass’s significance, prognosis, and appropriate management.

## Data Availability

The data presented in this study are available on request from the corresponding author due to privacy reasons.

## References

[1] Tower-Rader A., Kwon D. (2017). Pericardial Masses, Cysts and Diverticula: A Comprehensive Review Using Multimodality Imaging. Prog. Cardiovasc. Dis..

[2] Usta H., Colak A., Aydin Y., Kaya U., Kocak H., Ceviz M., Aydin F., Jalalzai I. (2025). Cardiac and pericardial hydatid cysts: A single center analysis of 22 cases. BMC Cardiovasc. Disord..

[3] Van Beek E.J., Stolpen A.H., Khanna G., Thompson B.H. (2007). CT and MRI of pericardial and cardiac neoplastic disease. Cancer Imaging.

[4] Yared K., Baggish A.L., Picard M.H., Hoffmann U., Hung J. (2010). Multimodality imaging of pericardial diseases. JACC Cardiovasc. Imaging.

[5] Maisch B., Seferović P.M., Ristić A.D., Erbel R., Rienmüller R., Adler Y., Tomkowski W.Z., Thiene G., Yacoub M.H., Priori S.G. (2015). Guidelines on the diagnosis and management of pericardial diseases. Eur. Heart J..

[6] Khayata M., Alkharabsheh S., Shah N.P., Klein A.L. (2019). Pericardial Cysts: A Contemporary Comprehensive Review. Curr. Cardiol. Rep..

[7] Klein A.L., Abbara S., Agler D.A., Appleton C.P., Asher C.R., Hoit B., Hung J., Garcia M.J., Kronzon I., Oh J.K. (2013). American Society of Echocardiography clinical recommendations for multimodality cardiovascular imaging of patients with pericardial disease: Endorsed by the Society for Cardiovascular Magnetic Resonance and Society of Cardiovascular Computed Tomography. J. Am. Soc. Echocardiogr..

